# First‐Line Treatment of IGHV‐Unmutated Chronic Lymphocytic Leukemia: A Network Meta‐Analysis of Targeted and Chemoimmunotherapy Regimens

**DOI:** 10.1111/ejh.70191

**Published:** 2026-04-14

**Authors:** Santino Caserta, Enrica Antonia Martino, Danilo Lofaro, Ernesto Vigna, Antonella Bruzzese, Francesco Mendicino, Maria Eugenia Alvaro, Caterina Labanca, Eugenio Lucia, Virginia Olivito, Nicola Amodio, Fortunato Morabito, Valter Gattei, Massimo Gentile

**Affiliations:** ^1^ Hematology Unit, Department of Onco‐Hematology AO of Cosenza Cosenza Italy; ^2^ Department of Mathematics and Computer Science University of Calabria Rende Italy; ^3^ Department of Experimental and Clinical Medicine University of Catanzaro Catanzaro Italy; ^4^ AIL Sezione di Cosenza Italy; ^5^ Clinical and Experimental Onco‐Hematology Unit, Centro di Riferimento Oncologico di Aviano (CRO), IRCCS Aviano Italy; ^6^ Department of Pharmacy, Health and Nutritional Science University of Calabria Rende Italy

**Keywords:** Bruton tyrosine kinase inhibitors, first‐line therapy, *IGHV* unmutated CLL, network meta‐analysis, PRISMA guidelines, progression‐free survival, venetoclax‐based therapy

## Abstract

Immunoglobulin heavy chain variable region‐unmutated (*IGHV*‐U) chronic lymphocytic leukemia (CLL) represents a biologically aggressive subgroup with limited responsiveness to chemoimmunotherapy (CIT). To clarify the comparative effectiveness of available frontline options, we conducted a comprehensive Bayesian network meta‐analysis of randomized clinical trials including more than 4500 *IGHV*‐U patients. Targeted therapies consistently outperformed CIT backbones, confirming the minimal benefit of cytotoxic approaches in this population. Acalabrutinib‐based regimens, either as monotherapy or combined with obinutuzumab, emerged as the most effective strategies for progression‐free survival, followed by other BTK inhibitors and venetoclax‐based combinations. Chlorambucil‐ and Fludarabine‐containing regimens ranked lowest. The fixed‐duration venetoclax‐obinutuzumab regimen also demonstrated strong efficacy, though estimates were less precise due to a smaller evidence base. Overall, heterogeneity was low, model fit was robust, and no statistical evidence was detected. These findings support targeted agents as the preferred first‐line treatment for *IGHV*‐U CLL and provide a quantitative framework to guide regimen selection while highlighting the need for head‐to‐head trials and long‐term follow‐up to optimize treatment sequencing.

## 1 Introduction

Chronic lymphocytic leukemia (CLL) is a mature B‐cell malignancy accounting for approximately one‐quarter of non‐Hodgkin lymphomas (NHL) [1, 2, 3]. Data from the latest SEER registry estimate an age‐adjusted incidence of about 4.9 cases per 100 000 individuals per year, confirming CLL as one of the most frequent hematological neoplasms in Western populations. Cytogenetically, CLL is defined by large chromosomal imbalances, such as deletions in 13q14, 11q, or 17p, and trisomy 12, as well as recurrent gene mutations that drive clonal evolution and treatment resistance; overall, nearly 80% of patients harbor at least one of these alterations [2].

From a biological and clinical standpoint, patients with an unmutated *IGHV* (*IGHV*‐U) status represent a distinct subgroup characterized by more active B‐cell receptor (BCR) signaling, enhanced cellular proliferation, and frequent expression of unfavorable biomarkers such as CD38 and ZAP‐70 [4]. This molecular profile is associated with faster disease kinetics, shorter lymphocyte doubling time, and reduced treatment‐free survival [4].

Compared with mutated *IGHV* (*IGHV*‐M) cases, *IGHV*‐U patients experience markedly shorter progression‐free and overall survival, typically 1–5 and 3–10 years, respectively, versus 9–19 and 18–26 years in *IGHV*‐M cohorts. Moreover, *IGHV*‐U status has consistently been associated with inferior outcomes following chemoimmunotherapy, including Fludarabine‐Cyclophosphamide‐Rituximab (FCR) or Bendamustine‐Rituximab (BR), although these regimens are now rarely employed [5].

In accordance with the International Workshop on CLL (iwCLL) criteria, treatment initiation should be deferred until objective signs of disease activity emerge, such as worsening cytopenias due to bone marrow infiltration, symptomatic or progressive splenomegaly or lymphadenopathy, a ≥ 50% increase in lymphocyte count over 2 months, or constitutional symptoms [6].

Before initiating therapy, reassessment of TP53 integrity and *IGHV* status is essential to guide optimal treatment selection. For patients with *IGHV*‐U disease, chemotherapy‐based regimens are largely obsolete, as targeted agents show superior efficacy, durability, and safety.

Bruton tyrosine kinase (BTK) inhibitors, including Ibrutinib [7], Acalabrutinib [8], and Zanubrutinib [9] represent the preferred frontline therapy for most *IGHV*‐U patients, having demonstrated significant and sustained improvements in progression‐free survival (PFS) and overall survival (OS), regardless of TP53 mutational status. By irreversibly binding BTK and suppressing BCR signaling, these agents effectively inhibit CLL cell proliferation and survival, enabling durable disease control even in biologically high‐risk patients [10].

Second‐generation covalent BTK inhibitors, such as Acalabrutinib and Zanubrutinib, offer comparable efficacy to Ibrutinib with improved tolerability and reduced cardiovascular toxicity, making them preferred options for elderly or comorbid patients. Continuous BTK inhibition remains the standard therapeutic strategy; however, treatment discontinuation is generally discouraged outside clinical trials, as relapse commonly follows drug interruption.

A time‐limited alternative is venetoclax, a selective BCL2 inhibitor, combined with the anti‐CD20 monoclonal antibody Obinutuzumab. This fixed‐duration regimen achieves deep molecular remissions and measurable residual disease (MRD) negativity in a substantial proportion of patients, including those with *IGHV*‐U [2, 11]. The choice between continuous BTK inhibition and fixed‐duration Venetoclax‐based therapy should be individualized, considering patient preference, comorbidities, cardiovascular risk, and adherence.

For these reasons, we performed a meta‐analysis integrating all randomized clinical trials evaluating first‐line treatment of *IGHV* unmutated CLL with the aim of providing a hierarchical comparison of contemporary therapeutic regimens.

## 2 Materials and Methods

### 2.1 Study Design and Search Strategy

This systematic review and network meta‐analysis (NMA) was conducted in accordance with the Preferred Reporting Items for Systematic Reviews and Meta‐Analyses (PRISMA) guidelines and was not registered in PROSPERO [12].

An extensive literature search was performed across PubMed, Scopus, and Embase, covering all records available up to December 2025. The search strategy combined terms related to Chronic lymphocytic leukemia (CLL) and *IGHV*‐U. Keywords and Medical Subject Headings (MeSH) included “IGHV unmutated CLL,” “CLL first‐line therapy,” “meta‐analysis,” “PRISMA guidelines,” “progression‐free survival,” and related synonyms [13]. The details about the search strategy are provided in the Supporting Information.

### 2.2 Inclusion and Exclusion Criteria

Studies were eligible if they met the following criteria: (i) prospective, phase III randomized controlled trials (RCTs); (ii) reporting outcomes stratified by *IGHV*‐U status; and (iii) providing quantitative measures of association for progression‐free survival (PFS), including adjusted hazard ratio (HR) with corresponding 95% confidence intervals (CIs).

Phase II studies, single‐arm trials were excluded (*n* = 100). Of the 34 studies that fulfilled the primary inclusion criteria, 19 were excluded due to incomplete or missing information regarding *IGHV*‐U subgroups.

### 2.3 Statistical Analysis

We conducted a Bayesian contrast‐based random‐effects Network Meta‐Analysis (NMA) to compare first‐line treatments in *IGHV*‐U CLL. The primary outcome was PFS HR. Multi‐arm trials were modelled, accounting for within‐study correlation [14]. The Bayesian framework was selected given the relatively sparse structure of the evidence network, where most treatment contrasts were informed by a single study. In this setting, hierarchical modeling allows coherent propagation of uncertainty across direct and indirect comparisons and stabilizes estimation of between‐study heterogeneity [15].

For basic treatment effects, we used weakly informative Normal priors. Between‐study heterogeneity was modeled on the log‐HR scale with a Uniform distribution prior for the SD (τ); robustness was explored using a half‐Normal prior for τ [16]. Four parallel Markov chains were run (50 000 iterations each; 5000 burn‐in; thinning interval = 2). Convergence was assessed visually and via the Gelman‐Rubin statistic (*R̂* ≤ 1.05). Model adequacy was evaluated using posterior mean residual deviance and the deviance information criterion (DIC).

Full model specification, including likelihood, parameterization, and prior distributions, is provided in the Supporting Information.

Relative effects are reported as posterior medians with 95% Credible Intervals (CrI; 2.5th–97.5th percentiles). A forest plot displays pooled posterior HRs versus Chlorambucil (Clb) as reference. Treatment ranking was summarized by the surface under the cumulative ranking curve (SUCRA), derived from the posterior rank distribution at each MCMC iteration; we report median SUCRA with 95% CrI and provide a complete pairwise league table [17].

Consistency was examined locally through node‐splitting analysis, comparing direct, indirect, and overall network estimates (each with 95% CrI), and globally through the design‐by‐treatment interaction test. Sensitivity analyses included: alternative τ priors (half‐Normal) and leave‐one‐treatment‐out NMAs to assess the influence of individual regimens on treatment rankings (Table 1).

**TABLE 1:** Surface under the cumulative ranking curve (SUCRA) values for each treatment.

Treatment	SUCRA	95% CrI	Broad treatment class
Acala + Obinu	0.952	(0.667–1.000)	BTKi (2nd gen.) + mAb
Acala	0.888	(0.500–1.000)	BTKi (2nd gen.)
Ibr + Obinu	0.82	(0.444–1.000)	BTKi (1st gen.) + mAb
IVO	0.801	(0.444–1.000)	BTKi (1st gen.) + anti‐BCL2 + mAb
Acala + Ven + Obinu	0.727	(0.278–1.000)	BTKi (2nd gen.) + anti‐BCL2 + mAb
Ibr + Ven	0.738	(0.500–0.944)	BTKi (1st gen.) + anti‐BCL2
Ven + Obinu	0.661	(0.444–0.833)	anti‐BCL2 + mAb
Ibr	0.587	(0.333–0.833)	BTKi (1st gen.)
Zanu	0.565	(0.222–0.944)	BTKi (2nd gen.)
Acala + Ven	0.528	(0.111–0.889)	BTKi (2nd gen.) + anti‐BCL2
R‐Ibr	0.467	(0.222–0.778)	BTKi (1st gen.) + mAb
FCR/BR	0.419	(0.111–0.722)	CHT + mAb
Obinu + Clb	0.346	(0.167–0.611)	CHT + mAb
R‐Ven	0.313	(0.056–0.667)	anti‐BCL2 + mAb
FCR	0.199	(0.056–0.444)	CHT + mAb
BR	0.169	(0.000–0.444)	CHT + mAb
R‐Clb	0.165	(0.000–0.500)	CHT + mAb
FC	0.107	(0.000–0.444)	CHT
Clb	0.049	(0.000–0.278)	CHT

*Note*: Values are posterior medians with 95% CrI, obtained by ranking treatments at each MCMC iteration and transforming ranks to SUCRA. Scale: 0–1 (higher = better).

Abbreviations: BTKi: BTK inhibitor; CHT: chemotherapy; mAb: monoclonal antibody.

All analyses were performed in R 4.5.1, using the gemtc package (JAGS backend via rjags).

## 3 Results

### 3.1 Study Selection

**FIGURE 1:**
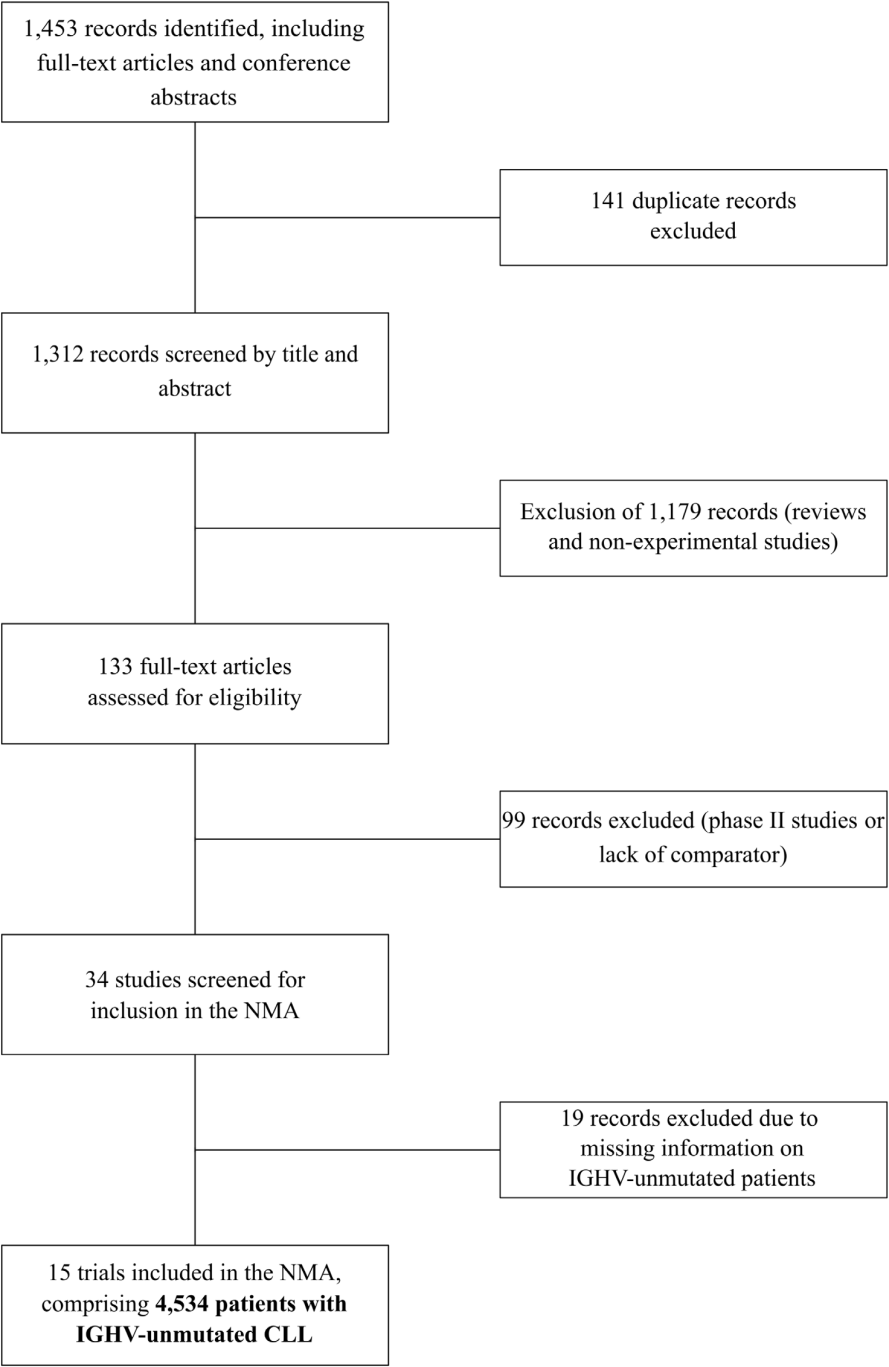
PRISMA 2020 flow diagram of study selection.

A total of 1453 records were initially identified through database searches, including full‐text articles and conference abstracts. After removal of 141 duplicates, 1312 unique studies were screened by title and abstract. Of these, 1179 records were excluded as they consisted of reviews, commentaries, or non‐experimental designs. The remaining 133 full‐text articles were assessed for eligibility according to predefined inclusion criteria; 99 were excluded for not meeting the inclusion criteria, and 19 were excluded because they did not report data specific to *IGHV*‐U patients. Ultimately, 15 randomized controlled trials met all eligibility criteria and were included in NMA, representing a total of 4534 patients with *IGHV*‐U CLL (Figure 1) [18, 19, 20, 21, 22, 23, 24, 25, 26, 27, 28, 29, 30, 31].

### 3.2 Risk of Bias Assessment

**FIGURE 2:**
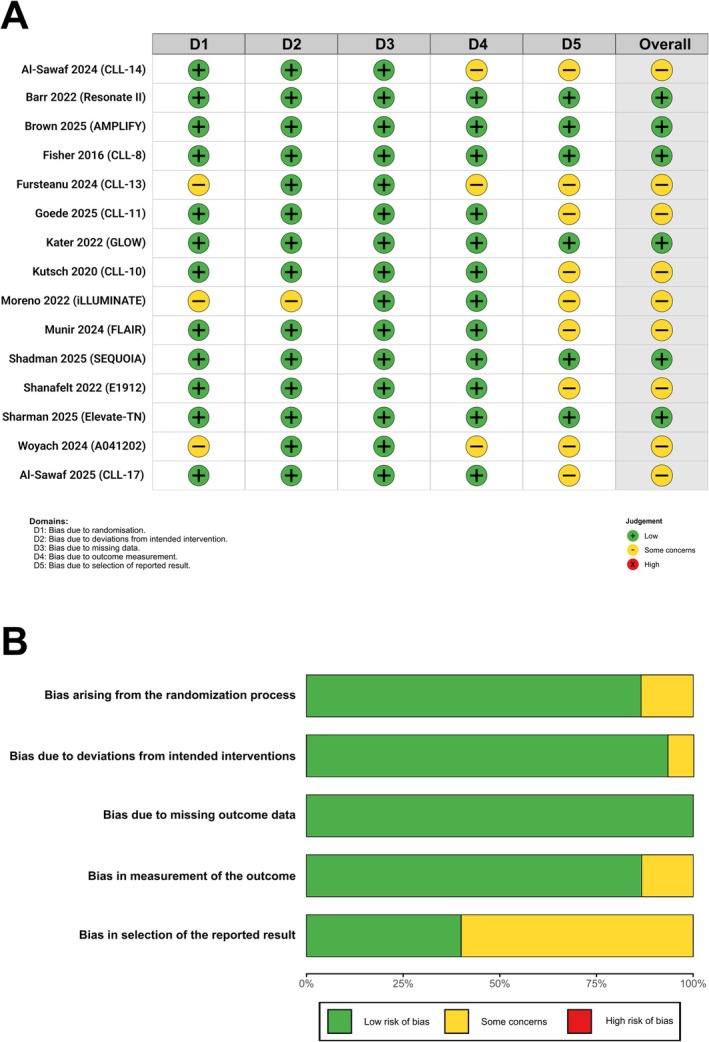
Risk‐of‐bias assessment of the included RCTs. (A) Traffic‐light plot of domain‐level RoB 2 ratings. (B) Stacked bar chart summarizing the proportion of trials rated Low risk (green), Some concerns (yellow), or High risk (red) across each domain.

Risk of bias was assessed using the Cochrane RoB2 tool (Figure 2) [32].

Across the 15 RCTs, no domain was judged at high risk of bias. Most trials were at low risk of randomization (D1), deviations from intended interventions (D2), missing outcome data (D3), and outcome measurement (D4). The domain with the most frequent concerns was D5 (selection of the reported result), particularly in studies where the analysis of *IGHV*‐U patients was not pre‐specified in the study protocol.

### 3.3 Network Meta‐Analysis of Progression‐Free Survival

The treatment network included 19 regimens connected through 15 RCTs (9 two‐arm, 5 three‐arm, 1 four‐arm). Each direct comparison was informed by a single study. Most regimens appeared in only one trial, whereas some “anchor” comparators, particularly Obinutuzumab‐Chlorambucil (Obinu+Clb; 5 studies) and Fludarabine‐Cyclophosphamide‐Rituximab (FCR; 4 studies), recurred across multiple trials.

Sample sizes varied substantially across regimens: FCR (*n* = 558), Obinu+Clb (*n* = 482), Bendamustine‐Rituximab (BR; *n* = 377), FCR/BR combined backbone (*n* = 303), Rituximab‐Ibrutinib (R‐Ibr; *n* = 280), and Venetoclax‐Obinutuzumab (Ven + Obinu; *n* = 422).

Overall, the network was adequately connected, enabling estimation of relative effects across all interventions, with higher precision expected for contrasts involving the larger comparator arms.

### 3.4 Comparative Efficacy

Between‐study heterogeneity was low (posterior median τ = 0.67, 95% CrI 0.11–2.35, log‐HR scale), with a network I^2^ = 5%. Model fit was satisfactory (DIC = 39.89). MCMC convergence was confirmed, with all Gelman‐Rubin *R̂* values < 1.05 and well‐mixed trace and density plots.

**FIGURE 3:**
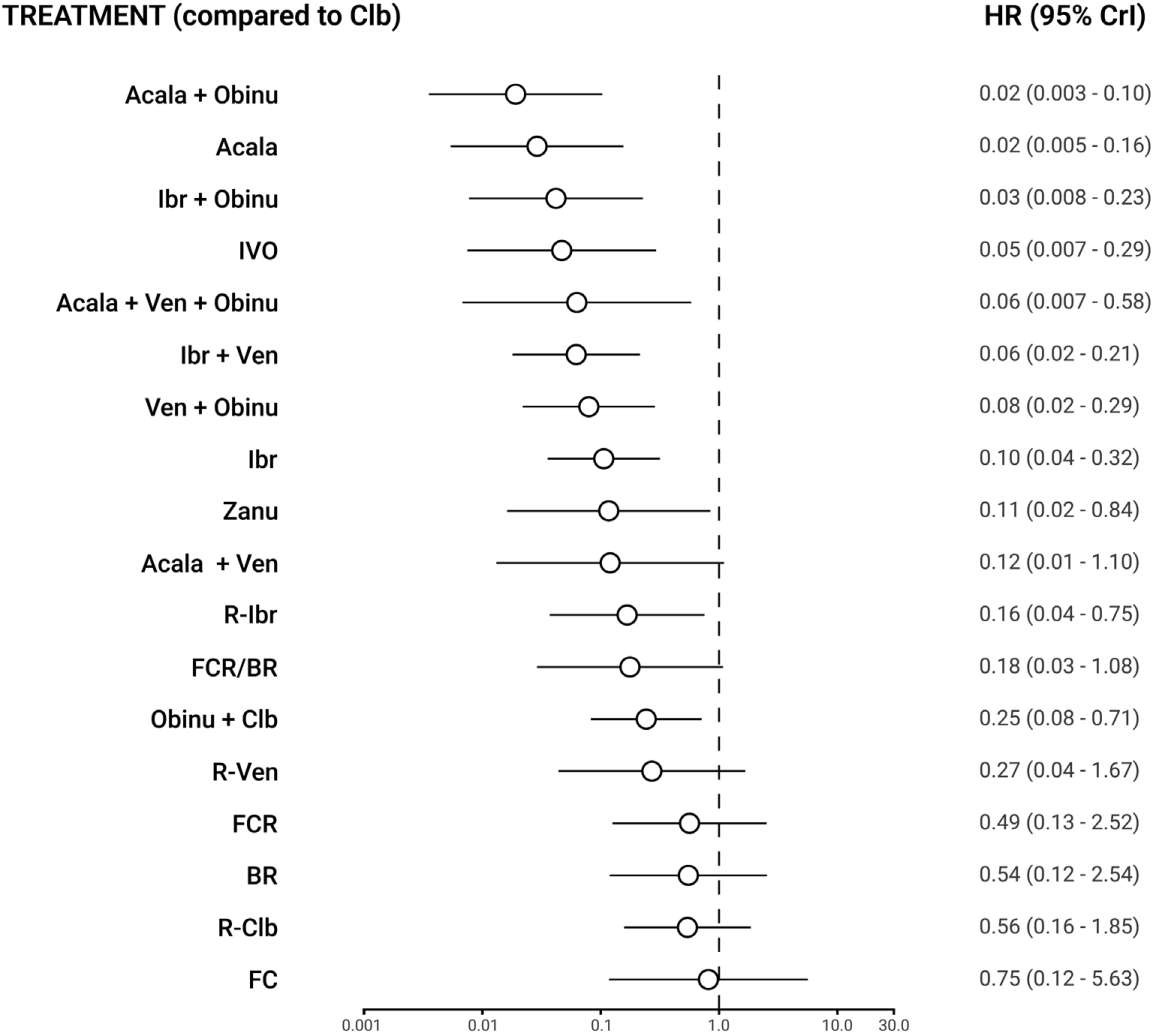
Forest plot of relative effects. Shown are HRs versus Chlorambucil (Clb) with 95% CrI. The x‐axis is on a logarithmic scale. White points denote posterior medians and bars the 2.5th–97.5th percentile; HR < 1 favors the treatment over CI.

Compared with Chlorambucil, targeted regimens showed large improvements in PFS (Figure 3). Acalabrutinib monotherapy (HR 0.02, 95% CrI 0.005–0.16) and Acalabrutinib plus Obinutuzumab (Acala+Obinu; HR 0.02, 95% CrI 0.003–0.1) ranked among the most effective regimens. Ibrutinib‐based combinations, including Ibrutinib plus Obinutuzumab (Ibr + Obinu; HR 0.03, 95% CrI 0.008–0.23), and Ibrutinib plus Venetoclax (Ibr + Ven; HR 0.06, 95% CrI 0.02–0.21), also marked superiority over Clb. Overall, BTK‐ and BCL2‐based targeted regimens demonstrated greater benefit than chemoimmunotherapy approaches.

The pairwise heatmap (Figure S1) and league table (Table S1) confirmed these findings. In particular, Acala+Obinu showed significantly greater efficacy than BR, FCR, R‐Clb, and Obinu+Clb.

Network consistency was assessed using node‐splitting analyses, which demonstrated no important inconsistency (all *p* values > 0.10; minimum *p* = 0.113), with close agreement between direct and indirect estimates for all assessed contrasts (Figure S2). Global tests were also supportive of consistency, as inconsistency models did not improve fit over the consistency model (ΔDIC = 0.109 for UME; 0.175 for the alternative parameterization).

### 3.5 Treatment Ranking

Treatment ranking aligned with the pairwise relative effects. Median SUCRA values were highest for Acala+Obinu (0.952) and Acala monotherapy (0.888), followed by Ibr + Obinu and IVO (0.820 and 0.801, respectively). In contrast, chemoimmunotherapy regimens ranked lowest (Clb 0.049 and FC 0.107, BR and FCR 0.16), in line with findings from the forest plot. Rankograms (Figure S3) supported the ranking stability across posterior distributions. Sensitivity analyses, using a leave‐one‐treatment‐out approach (Figure S4), showed only minor shifts in SUCRA values, confirming robustness of the ranking results.

Results were consistent under the alternative heterogeneity prior, with similar point estimates and treatment ranking. Credible intervals were slightly narrower, reflecting the more regularizing prior specification.

## 4 Discussion

In this NMA focusing on *IGHV*‐U CLL, we demonstrate that targeted agents, particularly second‐generation BTK inhibitors, either alone or combined with anti‐CD20 monoclonal antibodies, represent the most effective first‐line treatment options in terms of PFS [1]. Across 14 RCTs encompassing more than 4000 *IGHV*‐U patients, BTK‐ and BCL2‐directed regimens consistently and substantially outperformed CIT backbones, reinforcing the biological and clinical notion that *IGHV*‐U disease derives limited benefit from chemotherapy and requires mechanism‐driven therapeutic strategies.

Our findings reaffirm that *IGHV*‐U CLL is a high‐risk biological subset characterized by enhanced B‐cell receptor signaling and accelerated disease kinetics, translating clinically into inferior outcomes with CIT. In our analysis, Chlorambucil‐ and Fludarabine‐based regimens consistently ranked at the bottom of the treatment hierarchy, with effect sizes markedly inferior to targeted approaches. Conversely, Acalabrutinib, either as monotherapy or in combination with Obinutuzumab, yielded the most favorable PFS estimates and the highest SUCRA values, positioning it as the leading first‐line strategy within this population. These results were supported by robust model adequacy, low statistical heterogeneity, and an absence of meaningful inconsistency between direct and indirect comparisons [20, 30].

Acalabrutinib‐based regimens ranked among the most effective treatments in the network; however, differences relative to Ibrutinib‐based regimens were largely driven by indirect comparisons and should be interpreted cautiously. This apparent advantage is broadly consistent with emerging trial and real‐world evidence showing improved tolerability and at least comparable efficacy of second‐generation BTK inhibitors. While the network did not include head‐to‐head comparisons between Acalabrutinib and Zanubrutinib, the findings may be compatible with a potential class effect of covalent BTK inhibition as the backbone of therapy for *IGHV‐U* patients, irrespective of TP53 status [8], but do not allow firm conclusions in this regard. Moreover, combinations such as Ibrutinib‐Obinutuzumab and Ibrutinib‐Venetoclax also demonstrated highly favorable outcomes, suggesting that dual targeting of BCR signaling and apoptotic pathways may further enhance disease control, consistent with the biological rationale supporting synergistic pathway inhibition.

The fixed‐duration Venetoclax‐Obinutuzumab regimen also ranked among the most effective strategies, though with wider credible intervals reflecting a more limited evidence base. This underscores the growing relevance of time‐limited approaches capable of achieving deep remissions and MRD negativity in biologically adverse subgroups [10]. Phase 3 randomized trial CLL‐17, which enrolled patients with previously untreated CLL, demonstrated that two fixed‐duration regimens, venetoclax–obinutuzumab and venetoclax–ibrutinib, were non‐inferior to continuous ibrutinib. Three‐year PFS was 81.1% in the venetoclax–obinutuzumab arm, 79.4% in the venetoclax–ibrutinib arm, and 81% in the ibrutinib arm [33]. Nonetheless, continuous BTK inhibition remains the most extensively validated strategy in *IGHV*‐U CLL, supported by long‐term follow‐up available and durable disease control across multiple pivotal trials. Although the fixed‐duration regimens venetoclax–obinutuzumab and venetoclax–ibrutinib showed lower efficacy in terms of PFS compared with continuous targeted approaches, they retain a relevant therapeutic advantage by providing patients with a treatment‐free interval after completion of therapy, which represents a clinically meaningful benefit in terms of treatment burden and long‐term disease management.

In our meta‐analysis, we selected PFS as the primary endpoint rather than alternative outcomes such as time to next treatment (TTNT) because PFS is based on objective and standardized assessment criteria [6]. In particular, disease progression is defined according to internationally accepted guidelines, whereas the TTNT may be influenced by physician‐dependent clinical judgment.

**FIGURE 4:**
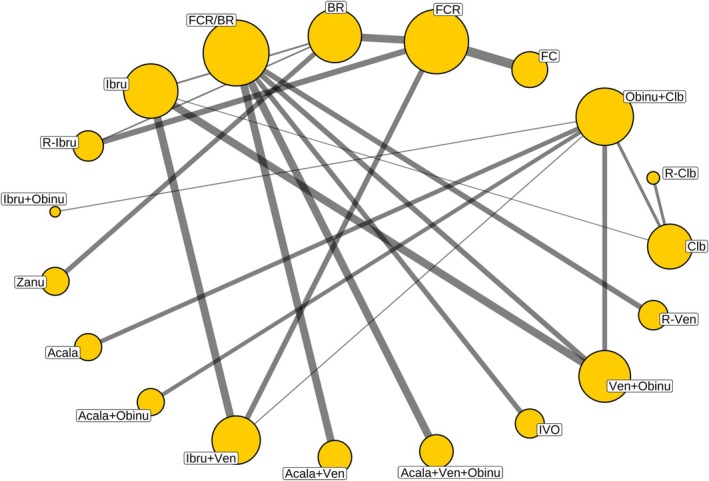
Network of treatment comparisons. Each node represents a treatment. The size of each node is proportional to the number of IGHV‐unmutated patients randomized to that treatment across the included studies, and the width of each edge is proportional to the number of IGHV‐unmutated patients contributing to the corresponding comparison. Each comparison is informed by a single trial.

Our analysis has several strengths, including comprehensive inclusion of all available randomized evidence, use of a Bayesian framework suited to estimating effects across a sparsely connected network, and rigorous assessment of both local and global consistency through node‐splitting and design‐by‐treatment interaction models (Figure 4).

However, several limitations must be acknowledged. First, most included trials did not prespecify *IGHV* subgroup analyses, introducing potential reporting bias, as reflected by the “some concerns” ratings for the RoB2 D5 domain. Most treatment contrasts were informed by single trials, which may limit the precision and generalizability of some estimates. Variability in trial populations, follow‐up duration, and outcome definitions may contribute to residual clinical heterogeneity not fully captured by the statistical model. PFS may be affected by differences in surveillance intensity across trials; in some experimental protocols, more frequent or systematic imaging assessments may lead to earlier detection of radiological progression, thereby artificially shortening PFS and introducing a potential detection bias that could disadvantage treatment arms subjected to more intensive monitoring. Moreover, the interpretation of PFS HRs across trials with heterogeneous follow‐up durations requires caution. Differences in follow‐up schedules, censoring patterns, and the validity of the proportional hazards assumption may contribute to variability in HR estimates, particularly in networks where most comparisons are informed by single studies. In this context, it should be acknowledged that the HR‐based comparisons are more assumption‐dependent than fixed‐time survival measures, such as landmark PFS estimates. However, PFS HRs were selected as the primary outcome because they represented the only effect measure consistently reported across the included trials. A further limitation concerns the magnitude of certain HR estimates in the network, particularly for Acalabrutinib‐based regimens, where some of the posterior median HRs are < 0.1. Estimates of this magnitude are unlikely to reflect only a pure biological effect, and are more plausibly explained by a combination of factors, including the indirect nature of all Acalabrutinib comparisons in the network, as well as differences in follow‐up duration and event accrual across trials. Importantly, despite these limitations, sensitivity analyses demonstrated a consistent direction of effect and stable treatment rankings, suggesting that the main comparative conclusions are robust to modeling assumptions. Clinical and biological heterogeneity within the subset of IGHV‐unmutated patients may have contributed to residual heterogeneity in the network.

Finally, although statistically significant differences emerge from this meta‐analysis, these findings should be interpreted with caution, as small variations in SUCRA values or ranking positions do not necessarily translate into clinically meaningful differences between treatments. Thus, statistical superiority should not be automatically equated with clinically relevant benefit.

Despite these limitations, the overall evidence strongly supports BTK inhibitor‐based therapy, particularly Acalabrutinib with or without Obinutuzumab as the most effective first‐line approach for patients with *IGHV*‐U CLL.

These results align closely with current iwCLL guidelines and further reinforce the obsolescence of CIT for this molecular subgroup [1].

As therapeutic decision‐making increasingly shifts toward individualized, biology‐driven strategies, our findings provide a quantitative framework to guide regimen selection and highlight the potential added value of combinatorial targeted approaches. Future head‐to‐head trials and long‐term follow‐up will be crucial to clarify the optimal sequencing of BTK and BCL2 inhibition and to determine whether time‐limited combinations can achieve durable disease control comparable to continuous therapy in *IGHV*‐U disease.

## 5 Conclusions

In this comprehensive network meta‐analysis of randomized clinical trials evaluating first‐line treatments for *IGHV*‐U CLL, targeted agents decisively outperformed chemoimmunotherapy, reinforcing the biological rationale that *IGHV*‐U disease derives minimal benefit from cytotoxic approaches [18, 19, 20, 21, 22, 23, 24, 25, 26, 27, 28, 29, 30, 31].

Acalabrutinib‐based regimens emerged as the most effective strategies in terms of PFS, followed by other BTK inhibitors and Venetoclax‐based combinations. These findings support the use of mechanism‐driven therapies as the preferred standard of care for *IGHV*‐U patients, irrespective of age or comorbidity.

Although evidence for newer combination strategies remains heterogeneous and long‐term survival outcomes are still maturing, our results provide a robust comparative framework to inform therapeutic decision‐making in this high‐risk biological subgroup. Future head‐to‐head trials and extended follow‐up will be essential to refine treatment sequencing and to determine whether time‐limited targeted regimens can deliver durable disease control comparable to continuous therapy.

## Author Contributions

All authors contributed to the manuscript and were involved in revisions and proofreading. All authors approved the submitted version.

## Funding

The authors have nothing to report.

## Conflicts of Interest

The authors declare no conflicts of interest.

## Supporting information


**Figure S1:** Relative effect matrix. Upper‐triangle heatmap of pairwise HRs. Cells report posterior medians for the row versus the column treatment; shading encodes the HR on a (log‐scaled) gradient centered at HR = 1. Values are shown only when the 95% CrI excludes 1 (HR < 1 favors the row treatment).

**Figure S2:** Node‐splitting results. Direct, indirect, and network estimates for selected treatment contrasts, reported as log(HR) with 95% CrI (points = posterior medians, bars = 2.5th‐97.5th percentiles). Direct and indirect estimates are contrasted to assess local inconsistency. No important inconsistency was detected: all *p* values ranged from 0.38 to 0.87.

**Figure S3:** Rankogram. For each treatment, stacked bars showing the posterior probability of attaining each possible rank based on the PFS (Rank 1 = best, Rank 19 = worst).

**Figure S4:** Stability of treatment ranking in leave‐one‐treatment‐out analyses. Box plots show the SUCRA values distribution in the sequential NMAs, excluding one regimen each time. Red points represent base SUCRA values.

**Table S1:** Full pairwise comparison table from the Bayesian NMA. Each cell displays the posterior median HR for the *column* treatment vs. the *row* treatment, with 95% CrIs. Values < 1 indicate benefit of the column treatment.

## Data Availability

Data sharing not applicable to this article as no datasets were generated or analysed during the current study.
