# Supplementary figures and images for: First‐Line Treatment of IGHV‐Unmutated Chronic Lymphocytic Leukemia: A Network Meta‐Analysis of Targeted and Chemoimmunotherapy Regimens
Source: Eur J Haematol. 2026 Apr 14;117(2):375–83. doi: 10.1111/ejh.70191 (PMC13326791; doi:10.1111/ejh.70191)

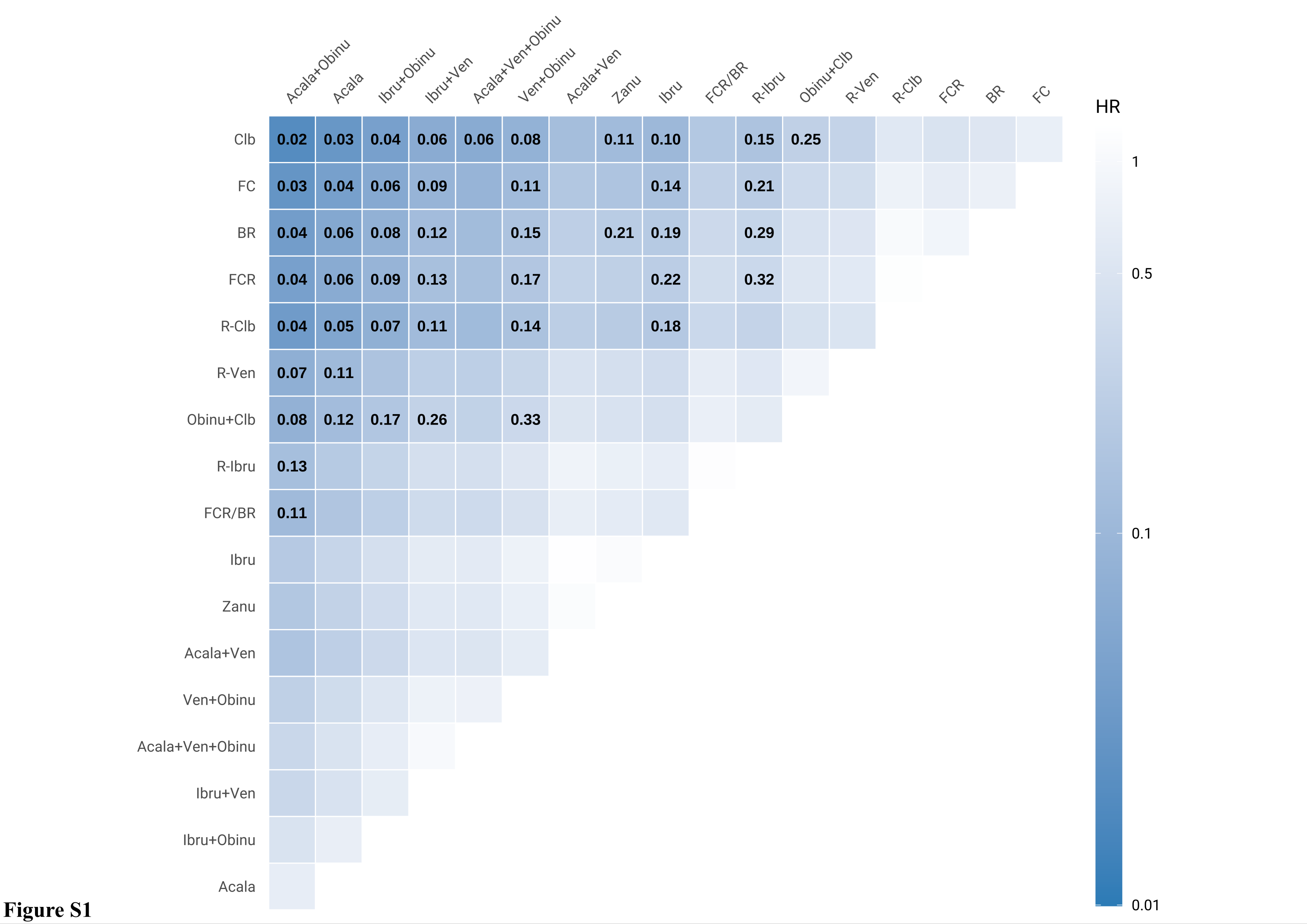

Supplement: Supplementary file 1 — Figure S1: Relative effect matrix. Upper‐triangle heatmap of pairwise HRs. Cells report posterior medians for the row versus the column treatment; shading encodes the HR on a (log‐scaled) gradient centered at HR = 1. Values are shown only when the 95% CrI excludes 1 (HR < 1 favors the row treatment). [file EJH-117-375-s005.tiff]

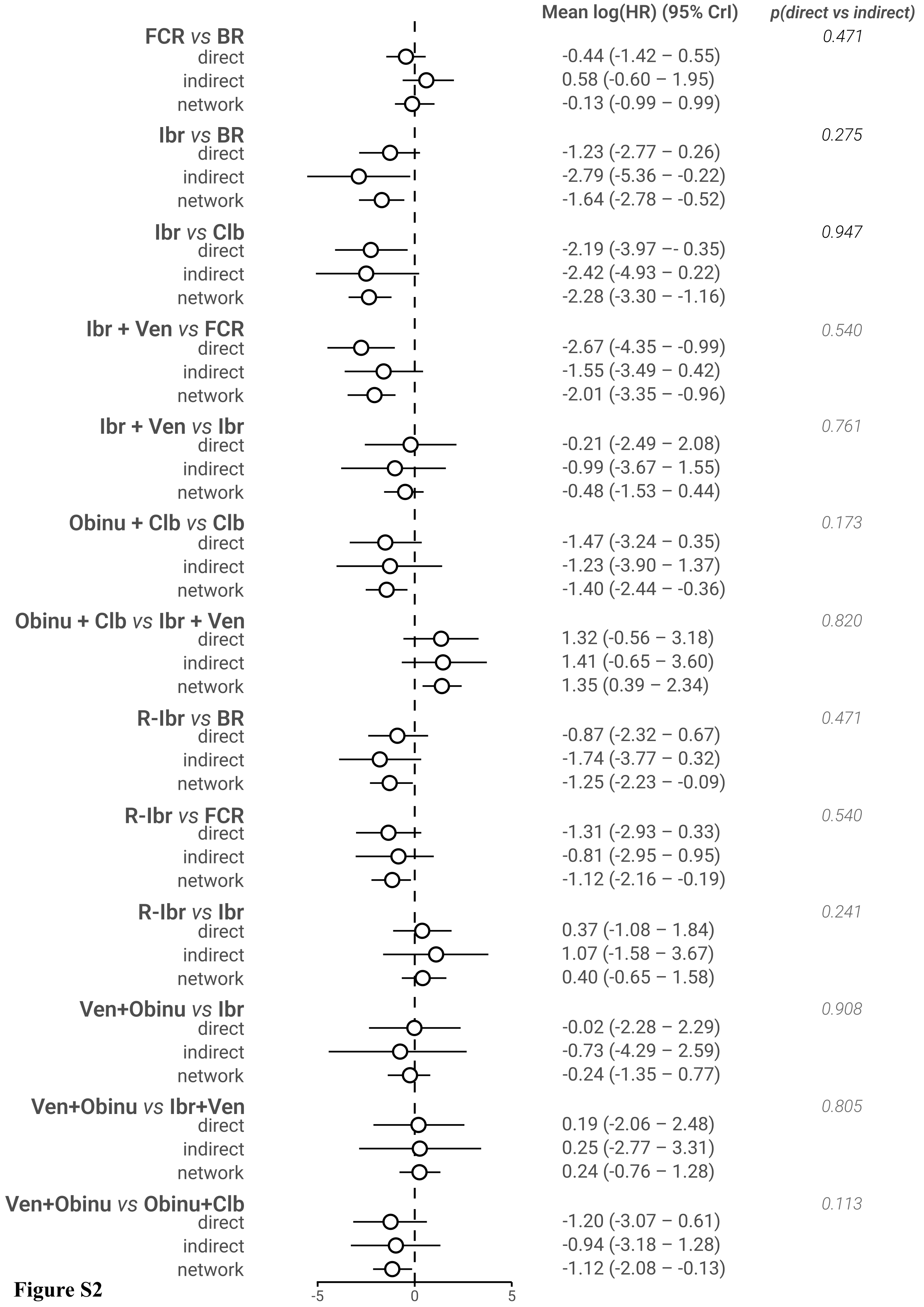

Supplement: Supplementary file 2 — Figure S2: Node‐splitting results. Direct, indirect, and network estimates for selected treatment contrasts, reported as log(HR) with 95% CrI (points = posterior medians, bars = 2.5th‐97.5th percentiles). Direct and indirect estimates are contrasted to assess local inconsistency. No important inconsistency was detected: all p values ranged from 0.38 to 0.87. [file EJH-117-375-s004.tiff]

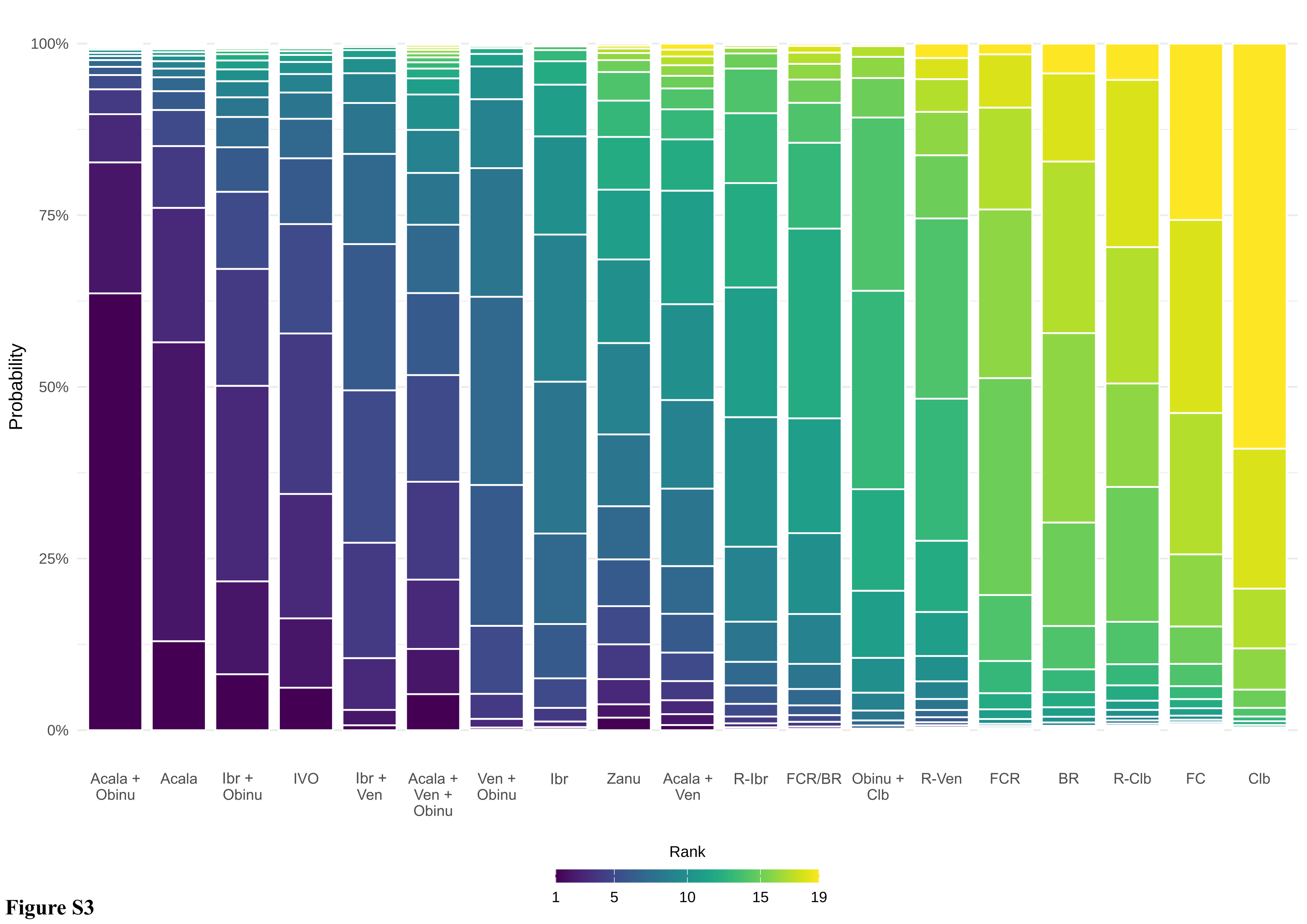

Supplement: Supplementary file 3 — Figure S3: Rankogram. For each treatment, stacked bars showing the posterior probability of attaining each possible rank based on the PFS (Rank 1 = best, Rank 19 = worst). [file EJH-117-375-s001.tiff]

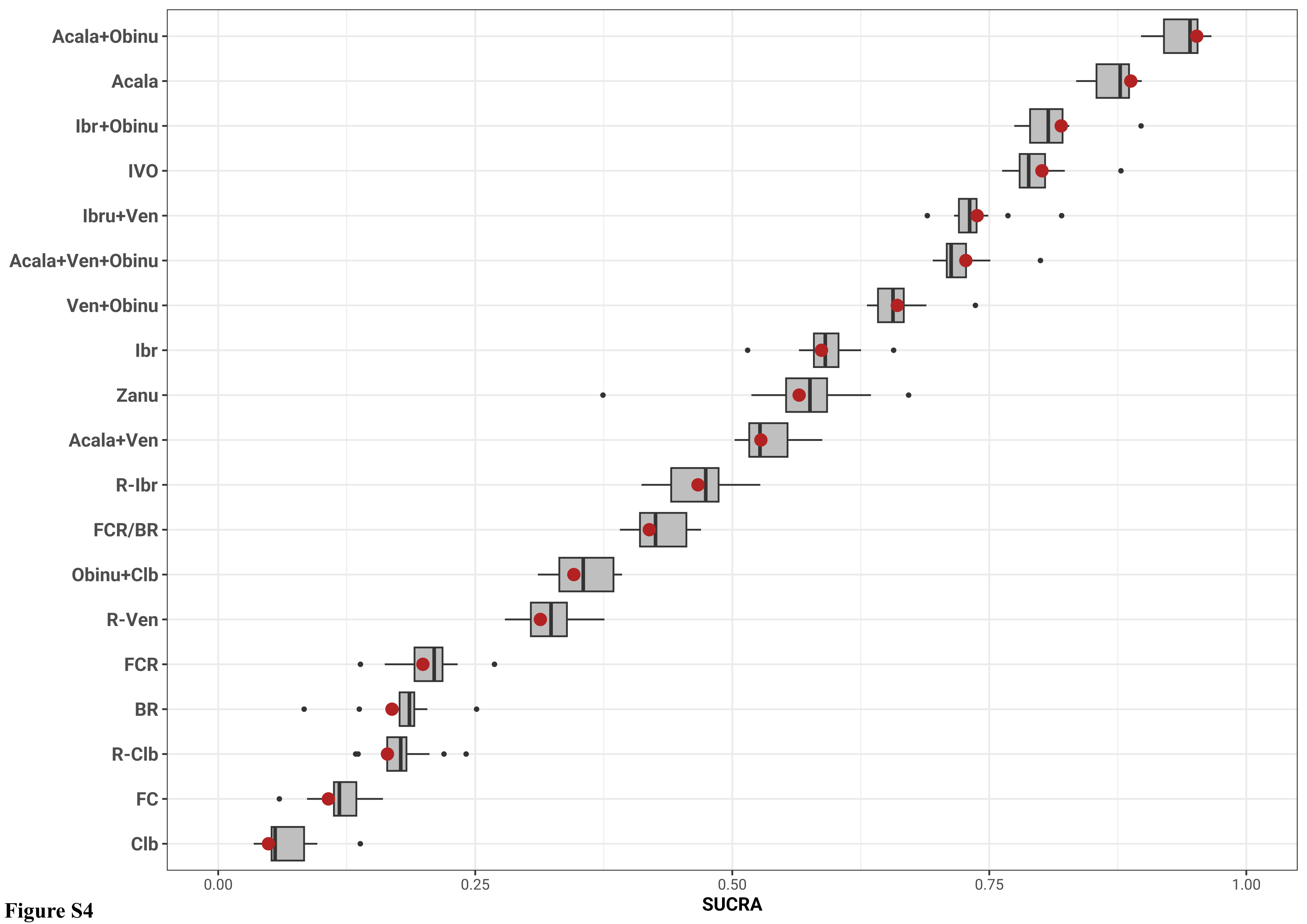

Supplement: Supplementary file 4 — Figure S4: Stability of treatment ranking in leave‐one‐treatment‐out analyses. Box plots show the SUCRA values distribution in the sequential NMAs, excluding one regimen each time. Red points represent base SUCRA values. [file EJH-117-375-s003.tiff]
