# Supplementary material for: First‐Line Treatment of IGHV‐Unmutated Chronic Lymphocytic Leukemia: A Network Meta‐Analysis of Targeted and Chemoimmunotherapy Regimens
Source: Eur J Haematol. 2026 Apr 14;117(2):375–83. doi: 10.1111/ejh.70191 (PMC13326791; doi:10.1111/ejh.70191)
Supplement: Supplementary file 5 — Table S1: Full pairwise comparison table from the Bayesian NMA. Each cell displays the posterior median HR for the column treatment vs. the row treatment, with 95% CrIs. Values < 1 indicate benefit of the column treatment. [file EJH-117-375-s002.docx]

**SUPPLEMENTARY APPENDIX**

**First-Line Treatment of IGHV-Unmutated Chronic Lymphocytic Leukemia Patients: A Network Meta-Analysis of Targeted and Chemoimmunotherapy Regimens**

Santino Caserta^1#^, Enrica Antonia Martino^1#^, Danilo Lofaro^2^, Ernesto Vigna^1^, Antonella Bruzzese^1^, Francesco Mendicino^1^, Maria Eugenia Alvaro^1^, Caterina Labanca^1^, Eugenio Lucia^1^, Virginia Olivito^1^, Nicola Amodio^3^, Fortunato Morabito^4*^, Valter Gattei^5*^, Massimo Gentile^1,6*^.

^1^Hematology Unit, Department of Onco-Hematology, AO of Cosenza, Cosenza, Italy; ^2^Department of Mathematics and Computer Science, University of Calabria, 87036 Rende, Italy; ^3^Department of Experimental and Clinical Medicine, University of Catanzaro, Catanzaro, Italy; ^4^AIL Sezione di Cosenza, Italy; ^5^Clinical and Experimental Onco-Hematology Unit, Centro di Riferimento Oncologico di Aviano (CRO), IRCCS, Aviano, Italy^; 6^Department of Pharmacy, Health and Nutritional Science, University of Calabria, Rende, Italy

^#^These authors contributed equally as first authors.

^*^These authors contributed equally as last authors.

**Correspondence**: Massimo Gentile, MD, Hematology Unit, AO of Cosenza, Italy; 87100 Cosenza, Italy; viale della Repubblica snc, e-mail: massimo.gentile@unical.it; ph: +39-0984-681329; fax: +39-0984-681329; Valter Gattei, MD, Clinical and Experimental Onco-Hematology Unit, Centro di Riferimento Oncologico di Aviano (CRO), IRCCS, Aviano, Italy; 33081 Aviano (PN), Italy; Via Franco Gallini 2, e-mail:vgattei@cro.it; ph: +39 0434 659410; fax: +39 0434 659409.

**Author Contributions:** All authors contributed to the manuscript and were involved in revisions and proofreading. All authors approved the submitted version.

**Conflicts of Interest:** The authors declare that the research was conducted without any commercial or financial relationships that could be construed as a potential conflict of interest.

**Data availability statement:** Data sharing does not apply to this article, as no new data were generated or analyzed in this study.

**Supplementary Methods**

**Supplementary Figures Legend**

**Supplementary Table: 1**

**Supplementary Methods.**

*Bayesian Network Meta-Analysis Model Specification*

A Bayesian contrast-based random-effects network meta-analysis was conducted to estimate relative treatment effects on progression-free survival (PFS), expressed as log hazard ratios (log-HR).

Observed study-specific treatment effects ($y_{ij}$) and corresponding standard errors ($\sigma_{ij}$) were modeled using a normal likelihood:

$$y_{ij}\sim\mathcal{N}(\delta_{ij},\sigma_{ij}^{2})$$

Treatment effects were parameterized using basic parameters $d_{k}$, with one treatment set as reference ($d_{1}=0$). Relative effects within each study were expressed as contrasts between treatments. A random-effects model was used to account for between-study heterogeneity. Multi-arm trials were modeled by accounting for the induced correlation between treatment contrasts within the same study.

Prior distributions followed the default specification implemented in the *gemtc* R package. In particular, the between-study standard deviation was assigned:

$$\tau\sim\text{Uniform}(0,2.65926)$$

and the basic treatment effects were assigned:

$$d_{k}\mathcal{\sim N}(0,{39.89}^{2})$$

where 2.65926 corresponds to the internally estimated outcome-measure scale (*om.scale*), defined as the maximum absolute observed treatment contrast across studies, used to calibrate weakly informative priors on the outcome scale, and 39.89 is derived as $15\times\text{om.scale}$.

A sensitivity analysis was performed by replacing the default uniform prior on the between-study standard deviation with a half-normal prior on the standard deviation scale:

$$\tau\sim\text{Half-Normal}(0,{1.32963}^{2})$$

where the scale parameter corresponds to $0.5\times\text{om.scale}$. All other model specifications were unchanged.

*Search strategy*

Equivalent search strategies were adapted for each database using the appropriate controlled vocabulary and free-text terms.

Pubmed query:

("Leukemia, Lymphocytic, Chronic, B-Cell"[Mesh] OR "chronic lymphocytic leukemia"[tiab] OR "chronic lymphocytic leukaemia"[tiab] OR "CLL"[tiab] OR "B-cell chronic lymphocytic leukemia"[tiab] OR "B-CLL"[tiab] ) AND

("Immunoglobulin Variable Region"[Mesh] OR "IGHV"[tiab] OR "unmutated IGHV"[tiab] OR "IGHV-unmutated"[tiab] OR "IGHV unmutated"[tiab]) AND

("first-line"[tiab] OR "frontline"[tiab] OR "front-line"[tiab] OR "initial treatment"[tiab] OR "initial therapy"[tiab] OR "previously untreated"[tiab] OR "treatment naive"[tiab] OR "treatment-naive"[tiab] OR "untreated"[tiab] OR "newly diagnosed"[tiab]) AND

("Randomized Controlled Trial"[Publication Type] OR "Clinical Trial, Phase III"[Publication Type] OR "Clinical Trial, Phase II"[Publication Type] OR "randomized controlled trial"[tiab] OR "randomised controlled trial"[tiab] OR "random*"[tiab] OR "phase 3"[tiab] OR "phase III"[tiab]) AND

("Survival"[Mesh] OR "Progression-Free Survival"[Mesh] OR "Treatment Outcome"[Mesh] OR "progression-free survival"[tiab] OR "PFS"[tiab] OR "event-free survival"[tiab] OR "hazard ratio"[tiab]).

**Supplementary Figures legend**

**Figure S1. Relative effect matrix.** Upper-triangle heatmap of pairwise HRs. Cells report posterior medians for the row versus the column treatment; shading encodes the HR on a (log-scaled) gradient centered at HR=1. Values are shown only when the 95% CrI excludes 1 (HR<1 favors the row treatment).

**Figure S2. Node-splitting results.** Direct, indirect, and network estimates for selected treatment contrasts, reported as log(HR) with 95% CrI (points = posterior medians, bars = 2.5th-97.5th percentiles). Direct and indirect estimates are contrasted to assess local inconsistency. No important inconsistency was detected: all p-values ranged from 0.38 to 0.87.

**Figure S3.** Rankogram. For each treatment, stacked bars showing the posterior probability of attaining each possible rank based on the PFS (Rank 1 = best, Rank 19 = worst).

**Figure S4.** Stability of treatment ranking in leave-one-treatment-out analyses. Box plots show the SUCRA values distribution in the sequential NMAs, excluding one regimen each time. Red points represent base SUCRA values.

**Table S1.** Full pairwise comparison table from the Bayesian NMA. Each cell displays the posterior median HR for the *column* treatment vs. the *row* treatment, with 95% CrIs. Values < 1 indicate benefit of the column treatment.

| **Acala+**  **Obinu** |  |  |  |  |  |  |  |  |  |  |  |  |  |  |  |  |  |  |
| --- | --- | --- | --- | --- | --- | --- | --- | --- | --- | --- | --- | --- | --- | --- | --- | --- | --- | --- |
| 0.66  (0.07-6.33) | **Acala** |  |  |  |  |  |  |  |  |  |  |  |  |  |  |  |  |  |
| 0.47  (0.02-10.59) | 0.71  (0.03-16.72) | **Ibr+**  **Obinu** |  |  |  |  |  |  |  |  |  |  |  |  |  |  |  |  |
| 0.45  (0.01-22.29) | 0.67  (0.01-33.79) | 0.94  (0.02-47.12) | **VOI** |  |  |  |  |  |  |  |  |  |  |  |  |  |  |  |
| 0.34  (0.02-6.94) | 0.51  (0.02-10.42) | 0.72  (0.04-14.49) | 0.76  (0.02-36.20) | **Ibr+**  **Ven** |  |  |  |  |  |  |  |  |  |  |  |  |  |  |
| 0.35  (0.00-28.22) | 0.52  (0.01-47.97) | 0.72  (0.01-65.63) | 0.77  (0.03-18.41) | 1.01  (0.01-88.39) | **Acala+Ven+**  **Obinu** |  |  |  |  |  |  |  |  |  |  |  |  |  |
| 0.27  (0.01-6.40) | 0.40  (0.02-9.38) | 0.56  (0.02-12.29) | 0.60  (0.06-5.74) | 0.78  (0.04-15.48) | 0.78  (0.03-19.99) | **Ven+**  **Obinu** |  |  |  |  |  |  |  |  |  |  |  |  |
| 0.17  (0.00-14.91) | 0.26  (0.00-23.23) | 0.36  (0.00-30.27) | 0.39  (0.02-8.53) | 0.51  (0.01-40.39) | 0.50  (0.05-4.37) | 0.66  (0.02-15.50) | **Acala+Ven** |  |  |  |  |  |  |  |  |  |  |  |
| 0.13  (0.00-8.09) | 0.20  (0.00-12.45) | 0.28  (0.00-18.02) | 0.29  (0.00-38.17) | 0.39  (0.01-12.15) | 0.38  (0.00-82.65) | 0.50  (0.01-34.53) | 0.76  (0.00-179.2) | **Zanu** |  |  |  |  |  |  |  |  |  |  |
| 0.13  (0.00-3.49) | 0.19  (0.01-5.27) | 0.27  (0.01-7.62) | 0.28  (0.00-16.37) | 0.37  (0.03-4.91) | 0.38  (0.00-39.34) | 0.48  (0.02-13.77) | 0.75  (0.01-81.19) | 0.97  (0.05-20.36) | **Ibr** |  |  |  |  |  |  |  |  |  |
| 0.12  (0.00-5.64) | 0.18  (0.00-9.05) | 0.25  (0.01-11.29) | 0.27  (0.03-2.45) | 0.35  (0.01-15.52) | 0.35  (0.04-3.37) | 0.45  (0.05-4.16) | 0.69  (0.08-7.09) | 0.91  (0.01-122.2) | 0.94  (0.02-55.68) | **FCR+**  **BR** |  |  |  |  |  |  |  |  |
| 0.09  (0.00-3.00) | 0.14  (0.00-4.46) | 0.20  (0.01-6.57) | 0.21  (0.00-14.15) | 0.27  (0.02-3.62) | 0.27  (0.00-31.74) | 0.35  (0.01-12.64) | 0.54  (0.00-66.41) | 0.71  (0.04-13.01) | 0.72  (0.09-5.45) | 0.78  (0.01-56.38) | **R-Ibr** |  |  |  |  |  |  |  |
| **0.08**  **(0.01-0.77)** | 0.12  (0.01-1.14) | 0.17  (0.02-1.49) | 0.18  (0.01-4.15) | 0.23  (0.03-1.83) | 0.23  (0.00-11.28) | 0.30  (0.03-2.88) | 0.46  (0.01-24.27) | 0.60  (0.02-22.10) | 0.63  (0.05-7.77) | 0.66  (0.03-16.45) | 0.86  (0.06-13.88) | **Obinu+**  **Clb** |  |  |  |  |  |  |
| 0.08  (0.00-3.93) | 0.12  (0.00-6.24) | 0.17  (0.00-7.94) | 0.18  (0.02-1.73) | 0.24  (0.01-10.27) | 0.23  (0.01-6.35) | 0.30  (0.03-2.75) | 0.46  (0.02-12.03) | 0.61  (0.00-71.77) | 0.63  (0.01-37.54) | 0.66  (0.07-6.35) | 0.86  (0.01-62.86) | 1.00  (0.04-24.08) | **R-Ven** |  |  |  |  |  |
| **0.03**  **(0.00-0.68)** | 0.05  (0.00-1.13) | 0.07  (0.00-1.49) | 0.07  (0.00-3.23) | 0.10  (0.01-1.66) | 0.09  (0.00-8.11) | 0.12  (0.01-2.62) | 0.19  (0.00-15.71) | 0.24  (0.01-11.41) | 0.25  (0.02-4.46) | 0.27  (0.01-11.69) | 0.35  (0.02-8.70) | 0.40  (0.04-3.59) | 0.41  (0.01-18.33) | **R-Clb** |  |  |  |  |
| **0.03**  **(0.00-0.87)** | 0.05  (0.00-1.26) | 0.07  (0.00-1.74) | 0.07  (0.00-4.15) | **0.09**  **(0.01-0.76)** | 0.09  (0.00-9.99) | 0.12  (0.00-3.24) | 0.18  (0.00-18.64) | 0.24  (0.01-4.00) | 0.25  (0.02-2.18) | 0.26  (0.00-14.39) | 0.34  (0.05-1.86) | 0.39  (0.03-4.81) | 0.40  (0.01-21.31) | 0.97  (0.04-20.14) | **FCR** |  |  |  |
| **0.03**  **(0.00-0.93)** | 0.04  (0.00-1.45) | 0.06  (0.00-2.03) | 0.06  (0.00-4.22) | 0.08  (0.01-1.05) | 0.08  (0.00-9.91) | 0.10  (0.00-3.45) | 0.16  (0.00-20.87) | 0.21  (0.02-2.05) | 0.21  (0.03-1.79) | 0.23  (0.00-15.65) | 0.30  (0.05-1.99) | 0.34  (0.02-5.49) | 0.34  (0.00-22.40) | 0.86  (0.04-19.71) | 0.87  (0.16-5.56) | **BR** |  |  |
| 0.02  (0.00-1.08) | 0.03  (0.00-1.78) | 0.04  (0.00-2.26) | 0.05  (0.00-4.77) | 0.06  (0.00-1.29) | 0.06  (0.00-9.75) | 0.08  (0.00-4.49) | 0.12  (0.00-20.16) | 0.16  (0.00-5.40) | 0.16  (0.01-3.41) | 0.17  (0.00-17.98) | 0.22  (0.01-3.83) | 0.26  (0.01-7.18) | 0.26  (0.00-26.68) | 0.64  (0.01-26.65) | 0.65  (0.07-5.86) | 0.76  (0.04-11.95) | **FC** |  |
| **0.02**  **(0.00-0.32)** | **0.02**  **(0.00-0.49)** | **0.03**  **(0.00-0.69)** | 0.04  (0.00-1.54) | **0.05**  **(0.00-0.60)** | 0.05  (0.00-3.91) | 0.06  (0.00-1.23) | 0.10  (0.00-8.06) | 0.12  (0.00-3.86) | 0.13  (0.02-1.04) | 0.14  (0.00-5.78) | 0.18  (0.01-2.56) | 0.21  (0.03-1.52) | 0.21  (0.00-9.12) | 0.51  (0.05-4.75) | 0.52  (0.04-7.76) | 0.59  (0.04-8.14) | 0.80  (0.03-29.27) | **Clb** |
